# Anterior dislocation of THA after Iliopsoas tenotomy in spinopelvic imbalance: a rare case report

**DOI:** 10.1186/s12891-020-03711-6

**Published:** 2020-11-07

**Authors:** Sun-Jung Yoon, Jong-Hyun Ko, Dean K. Matsuda

**Affiliations:** 1 Department of Orthopedic Surgery, Research Institute of Clinical Medicine, Jeonbuk National University-Biomedical Research Institute of Jeonbuk National University Hospital, Gunji-ro 20, Dukjin-gu, Jeonju, Jeonbuk 54907 Republic of Korea; 2 Premier Hip Arthroscopy, Marina Del Rey, CA USA

**Keywords:** Case report, Impingement, Iliopsoas tenotomy, Anterior dislocation, Total hip arthroplasty

## Abstract

**Background:**

Iliopsoas impingement is a complication of total hip arthroplasty that often manifests as groin pain during initial hip flexion. However, there are no reports of mechanical complications after iliopsoas tenotomy following total hip arthroplasty (THA).

**Case presentation:**

We present the case of a 64-year-old woman with degenerative lumbar kyphosis who developed anterior hip dislocations after arthroscopic iliopsoas tenotomy for anterior impingement following THA. She ultimately required revision of the acetabular cup.

**Conclusions:**

Arthroscopic tenotomy for refractory iliopsoas tendinopathy may be appealing because of its less invasive nature. However, caution should be exercised in the setting of significant cup malpositioning and/or spinopelvic imbalance to avoid iatrogenic anterior instability.

## Background

Iliopsoas impingement is a complication of total hip arthroplasty (THA), which often manifests as groin pain during initial hip flexion. Initial management commonly involves conservative treatment with non-steroidal anti-inflammatory drugs (NSAIDs), activity modification, and physical therapy. Failing that, ultrasound-guided injection into the sheath of the iliopsoas tendon is an effective diagnostic and therapeutic option.

The proposed pathogenesis of tendinosis is an induction by mechanical irritation or tenting of the iliopsoas tendon over the sharp edge of the acetabular component or bony margin during flexion and extension [1]. Ultrasound may reveal tendinosis with abnormal dynamic gliding of the iliopsoas muscle-tendon unit.

Iliopsoas recession is an option when more conservative measures fail. Trousdale et al. reported performing open tenotomy for iliopsoas tendinitis after THA [2]. More recently, the arthroscopic recession of the iliopsoas tendon has become available to address this impingement [3]. Therefore, endoscopic or arthroscopic tenotomy is generally effective if conservative treatment has failed. However, there are no reports of mechanical complications after iliopsoas tenotomy following THA. Moreover, the spinopelvic relationship has not been considered in any publication on arthroscopic iliopsoas recession. We present a case report of anterior dislocations after arthroscopic iliopsoas tenotomy for anterior impingement after THA in a patient with degenerative lumbar kyphosis.

## Case presentation

**Fig. 1:**
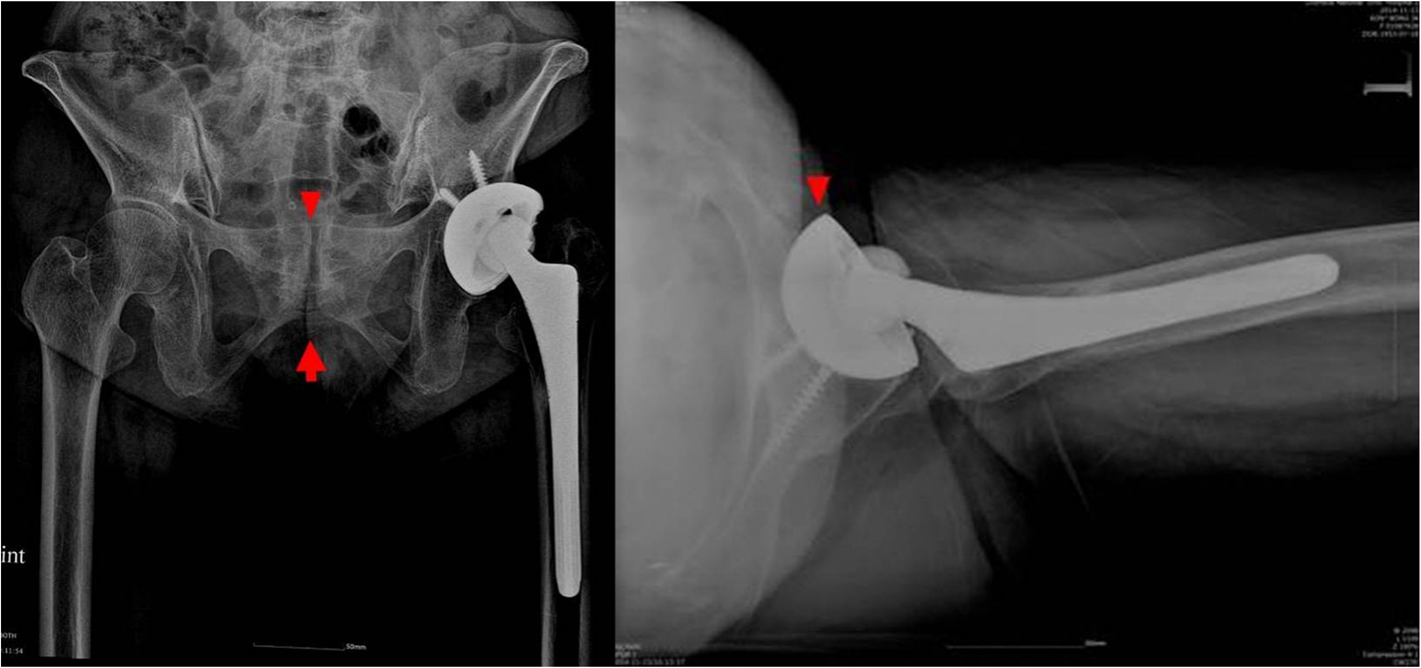
**a**. Standing pelvis anteroposterior radiographs show pelvic tilt upward due to degenerative lumbar kyphosis. The arrowhead indicates that the superior border of the symphysis pubis was located at the level of the third sacral vertebral body in the standing position. **b**. The cross-table lateral view shows that the anterior rim (arrowhead) of the acetabular component protrudes over the anterior wall of the acetabulum

**Fig. 2:**
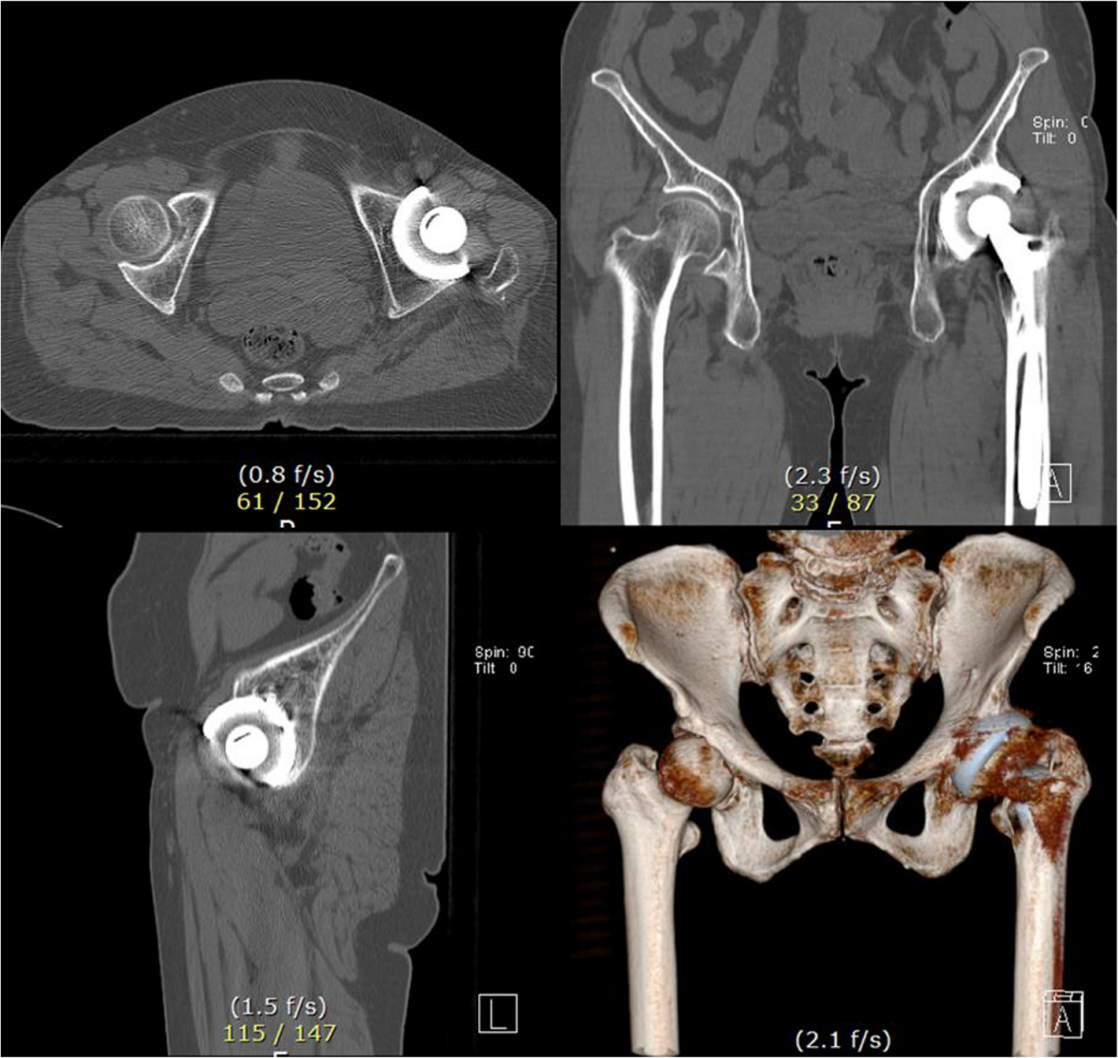
Cross-sectional computed tomography images and three-dimensional reconstruction images show a globally protruded acetabular component in the dysplastic acetabulum with a small volume

A 64-year-old female patient with prior THA presented with a seven-year history of groin pain and weakness during initial hip flexion. The THA was initially performed at a different hospital to address painful dysplastic osteoarthritis. Ipsilateral groin pain was presented soon after the THA. Moreover, stair climbing and exiting cars were aggravating activities. Pressure on the iliopsoas tendon of the groin was painful, especially during the Stinchfield test. A relatively large cup was implanted (58 mm in diameter) in the shallow hypovolemic acetabulum. Insufficient medialization was observed on the anteroposterior (AP) radiograph from the first outpatient clinic (Fig. 1). It was thought that periprosthetic joint infection could be the cause of the pain and was excluded using laboratory tests and ultrasound screening. The pelvis tilted upward posteriorly due to global sagittal malalignment associated with multiple spondylolistheses and degenerative kyphotic deformity in the lumbar spine. The upper border of the pubic symphysis was at the level of the third sacral vertebral body on a standing pelvic AP radiograph. The abduction angle of the acetabular component was 57.3°. On the axiolateral radiograph, the anteversion angle of the cup was 30.3°. The protrusion of the acetabular component was approximately 25 mm on the sagittal computed tomography image (Fig. 2).

NSAIDs and physical therapy failed to manage the pain. Multiple ultrasound-guided injections provided only transient analgesia. After conservative treatment failed, revision THA was offered as a treatment of choice, but she refused a revision arthroplasty because she was not satisfied with the index THA. Informed consent was obtained for arthroscopic tenotomy. It had been sufficiently explained that revision THA is necessary if there is no effect. Finally, she agreed to undergo arthroscopic iliopsoas tenotomy instead of a cup revision as an alternative minimally invasive manner instead of revision THA. Hip arthroscopy was performed using a standard traction device in the supine position. Under sufficient hip distraction confirmed via AP fluoroscopic imaging, we used the standard anterolateral viewing portal and a modified midanterior working portal.

**Fig. 3:**
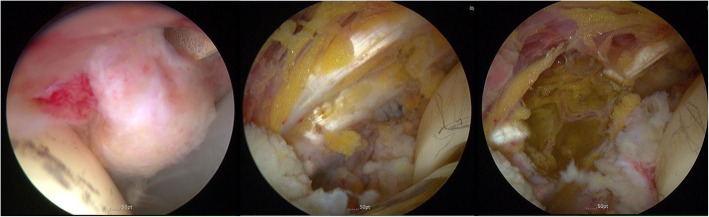
Arthroscopy shows psoas bursitis and iliopsoas tendinitis. Iliopsoas tenotomy was performed

**Fig. 4:**
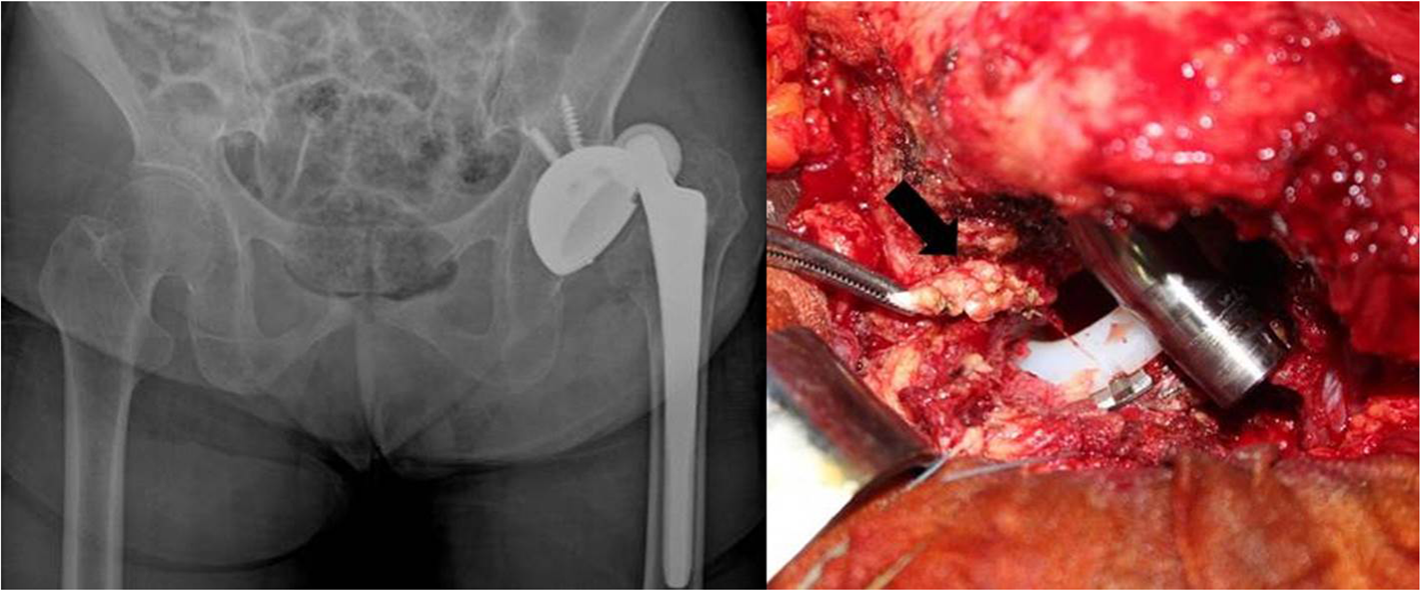
*A. anterior* dislocation occurred one month after arthroscopic tenotomy of the iliopsoas. B. Tenotomized stumps of the iliopsoas tendon. The arrow indicates a retracted iliopsoas tendon previously recessed at the level of the lesser trochanter

Sixty mmHg of fluid pressure was used with an intermittent increase up to 80 mmHg. After arthroscopic interportal pseudo-capsulotomy, psoas bursitis and iliopsoas tendinitis were observed near the acetabular cup overhang (Fig. 3). A bifid iliopsoas tendon was identified and tenotomized using a monopolar hook-shaped diathermy probe under endoscopic visualization without incident. Postoperatively, she was managed in a brace with physiotherapy. Her groin pain during initial hip flexion resolved immediately following arthroscopic iliopsoas tenotomy. She was delighted with the result and the minimally invasive surgery. One month postoperatively, she presented to the emergency department with an anterior dislocation of the THA after sitting on the floor in Buddha’s position (Fig. 4a). After closed reduction, the anterior apprehension test was positive. Hip bracing of the reduced THA was used as she refused further surgery. When reviewed three months after the arthroscopic tenotomy, she still complained of anterior instability and apprehension and subsequently experienced a second dislocation. She gave informed consent for revision surgery of the acetabular component. Revision THA was performed using the previous posterolateral approach. During revision surgery, tenotomized iliopsoas tendons were observed adjacent to the overhang of the protruding acetabular component (Fig. 4b).

**Fig. 5:**
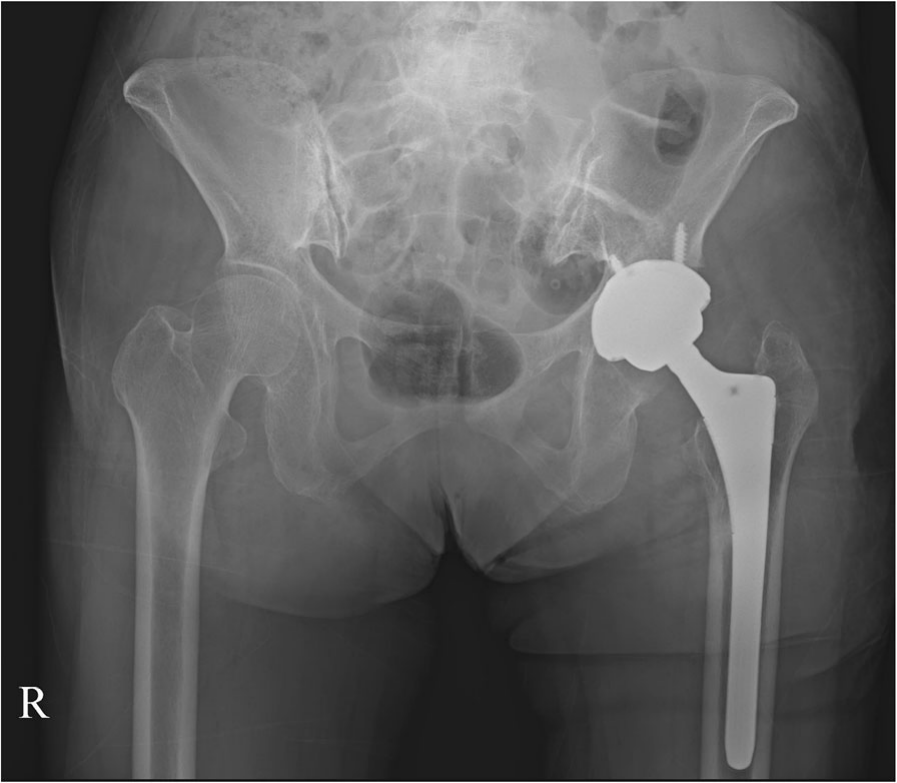
Postoperative hip anteroposterior radiograph. Revision total hip arthroplasty was performed only for the acetabular component using a 36-mm head. At 18 months, the patient had a negative Stinchfield test and was pleased with the outcome of no subsequent groin pain, flexion weakness, apprehension, or dislocation

**Fig. 6:**
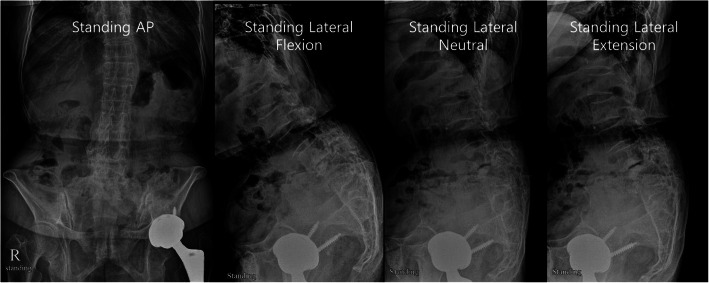
Standing anteroposterior (AP) and lateral radiography of the lumbar spine. The fifth lumbar vertebra is sacralized. On AP view, the obturator foramen of the pelvis is larger than that of a normal pelvis. On lateral views, multiple spondylolistheses of the third, fourth, and fifth lumbar vertebrae are observed, and the sacral slope is decreased due to posterior rotation of the sacrum and pelvis

**Fig. 7:**
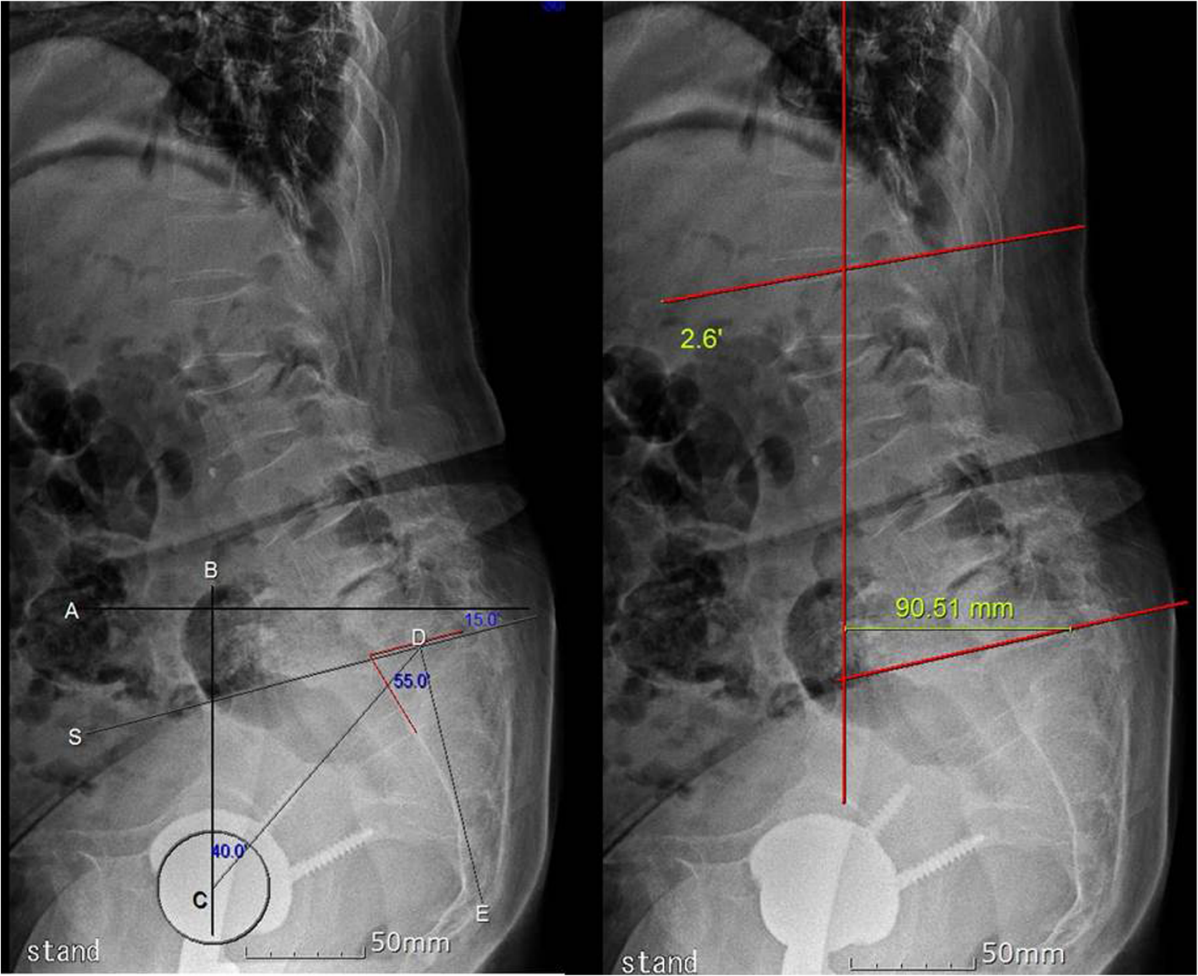
**a**. Pelvic parameters. The red line represents the anterior and superior border of the S1 body. Line A is the horizontal axis of the body. Line B is the vertical axis of the body. Line S is the continuation of the upper border of S1. Line E is the line perpendicular to line S. Point C is the center of the hip joint. Point D is the center of the upper border of S1. ∠BCD is a pelvic tilt (PT). ∠CDE is pelvic incidence. The angle between lines A and S is the sacral slope (SS). The decrease of SS and the increase of PT are shown to be associated with the global sagittal imbalance and degenerative lumbar kyphosis. PT is 40°, SS is 15°, and PI (pelvic incidence) is 55°. **b**. Lumbar lordosis and sagittal balance. SVA (sagittal vertical axis; C7 plumb line) traverses nine cm anterior to the posterosuperior corner of S1, and the L1-S1 Cobb’s angle is 2.6°

The acetabular component was explanted using a cup removal instrument (X-plant, Zimmer). The hip center was medialized to improve coverage of the cup by reaming toward the medial direction. The femoral component was retained. A porous-coated multihole cup was carefully implanted and fixed using multiple screws. A 36-mm delta ceramic head was used. After the multidirectional stability test was performed, the short external rotators were repaired using transosseous fixation. She recovered entirely by three months postoperatively. At 18 months postoperatively, she had a negative Stinchfield test, and she was pleased with the outcome of no subsequent groin pain, flexion weakness, apprehension, or dislocation (Figs. 5, 6 and 7).

## Discussion and conclusions

The iliopsoas muscle and tendon and iliocapsularis muscle complex stabilizes the dysplastic hip joint [4]. Furthermore, the hip capsule in general and the iliofemoral ligament, in particular, are important joint stabilizers. Arthroscopic femoroacetabular impingement surgery could accelerate osteoarthritic changes from potential microinstability in acetabular dysplasia, especially if the structures mentioned above are significantly compromised [5].

In THA, the iliopsoas tendon may also act as a last resort structure to prevent anterior dislocation, especially in the dysplastic acetabulum [4]. Physicians should distinguish whether the pain originates from the iliopsoas impingement itself or instability of the THA. Suboptimal anterior stability may occur with an unacceptable cup position when iliopsoas recession is needed or performed, especially in the setting of excessive acetabular component anteversion with insufficient medialization.

The sagittal alignment of the spine (lordosis or kyphosis) can alter the position of the pelvis and influence the pelvic parameters [6–8]. Lumbar lordosis is the angle between the upper endplate of L1 and the upper endplate of S1, and its normal ranges are 41 ± 11° in males and 46 ± 11° in females [9]. The pelvic tilt is the angle between the vertical axis and the line joining the middle of the S1 endplate with the center of the femoral heads, and the normal range is 13 ± 6° [10]. The sagittal alignment of the spine and the pelvic parameters are closely related. In this case, the lumbar lordosis was 2.6°, and the pelvic tilt was 40° after the revision of the THA, indicating that the global sagittal malalignment of the spine existed preoperatively and that, during the index operation, the acetabular cup could be placed in a more anteverted position. The sagittal alignment of the spine and the pelvic tilt is important radiographic factors of THA for the prevention of malpositioning of the acetabular cup and of postoperative groin pain. In this case report, acetabular dysplasia was replaced by a relatively large cup at the index THA. This procedure could theoretically treat the dysplastic and arthritic hip joints. However, this large cup irritated her iliopsoas tendon, causing intractable groin pain. Also, abnormal spinopelvic parameters, such as the pelvic tilt, could aggravate her groin pain.

The lumbar spine, pelvis, and hip form a complex mechanical unit in the surgery-naïve patient to compensate for limited posterior pelvic motion with greater hip flexion while seated. Similarly, patients with limited anterior pelvic motion compensate by hyperextending their hips in order to stand. In other words, changes in the spine and pelvis lead to changes at the hip. A patient with lumbar degenerative kyphosis has decreased lumbar lordosis due to muscle fatigue. Consequently, the compensatory posterior pelvic tilt could increase the anteversion and hyperextension of the hip joint. Therefore, the THA could be at risk for anterior dislocation [11]. Iliocapsularis muscle and iliopsoas tendon may act as an anterior stabilizer complex to prevent anterior subluxation in the dysplastic hip. The iatrogenic anterior instability induced by the capsulectomy, iliopsoas tenotomy, and partial resection of iliocapsularis muscle, by hip arthroscopy, is worsened by the posterior pelvic tilt increasing anteversion of the acetabulum. In the case of the native hip, partial flexion weakness may develop immediately after surgery when iliopsoas tenotomy is performed, and it is reported that it usually improves within 6 months. In the end, the lesson to be learned from this case report is that a careful approach to solving the underlying problem is the most important than minimal or least invasive surgery.

This case suggests that orthopedic surgeons need a comprehensive understanding of the tendon problems, instability, and complications associated with total hip arthroplasty that may occur after the spinopelvic imbalance affects the unacceptable cup position.

Iatrogenic anterior THA dislocation may occur with iliopsoas tenotomy in the absence of repair. Although arthroscopic tenotomy for refractory iliopsoas tendinopathy may be appealing because of its less invasive nature, caution should be exercised in the setting of significant cup malpositioning and/or spinopelvic imbalance to avoid iatrogenic anterior instability.

Table 1 Lessons from this case report

**Table:** 

✧ If a globally protruded malpositioned cup induces groin pain, including irritation of the iliopsoas tendon, cup revision might be preferable to prevent additional surgical intervention.	
✧ THA with a small femoral head or excessive pelvic compensation with degenerative lumbar kyphosis may increase the risk for dislocation.	
✧ Iliopsoas tendon recession with capsulectomy should be performed with caution in a patient with a spinopelvic imbalance.	
✧ The iliopsoas tendon and iliocapsularis muscle may act as a last resort anterior stabilizer to prevent anterior dislocation, especially in a THA with a malpositioned component and suboptimal stability.	

## Data Availability

Not applicable.

## Abbreviations

AP: Anteroposterior
NSAIDs: Non-steroidal anti-inflammatory drugs
THA: Total hip arthroplasty
IP: Iliopsoas
FAI: Femoroacetabular impingement
